# Redeployment of ophthalmologists in the United Kingdom during the Coronavirus Disease Pandemic

**DOI:** 10.1177/1120672120953339

**Published:** 2021-09

**Authors:** Christina Lim, Ian De Silva, George Moussa, Tahir Islam, Lina Osman, Huzaifa Malick, Sundeep Deol, Moheeb Youssef, Abdelsattar Farrag, Rehana Ashraf, Sreekala Burgula, Jonathan Thompson

**Affiliations:** 1 Department of Ophthalmology, University Hospitals of Leicester NHS Trust, Infirmary Square, Leicester, UK; 2 University Hospitals of Coventry and Warwickshire NHS Trust, Clifford Bridge Road, Coventry, UK; 3 University of Leicester, University Road, Leicester, UK

**Keywords:** Coronavirus, COVID-19, redeployment, intensive care unit, intensive treatment unit

## Abstract

**Background:**

During the current coronavirus (COVID-19) pandemic, some ophthalmologists across the United Kingdom (UK) have been redeployed to areas of need across the National Health Service (NHS). This survey was performed to assess aspects of this process including training & education, tasks expected, availability of personal protection equipment (PPE) used and the overall anxiety of ophthalmologists around their redeployment.

**Method:**

Online anonymous survey around the existing guidance on safe redeployment of secondary care NHS staff and PPE use by NHS England and Public Health England respectively. The survey was open to all ophthalmologists across the UK irrespective of their redeployment status.

**Findings:**

145 surveys were completed and returned during a 2-week period between 17th April 2020 and 1st May 2020, when 52% of ophthalmologists were redeployed. The majority of this group consisted of ophthalmologists in training (79%). 81% of those redeployed were assigned to areas of the hospital where patients with confirmed Coronavirus disease were being treated as inpatients. There was a statistically significant improvement in anxiety level following redeployment which was mainly attributed to the support received by staff within the redeployed area. 71% of the redeployed group were found to have sufficient PPE was provided for the area they worked in.

**Interpretation:**

This is the first national survey performed on redeployment of ophthalmologists in the UK. The study showed that ophthalmologists across all grades were able to contribute in most aspects of patient care. Anxiety of redeployment was reduced by prior training and good support in the redeployment area.

## Introduction

Since the announcement of the COVID-19 (coronavirus disease) pandemic by the World Health Organization (WHO), there have been dramatic changes to service provision across the National Health Service (NHS) in the United Kingdom (UK). As of 4th May 2020, there have been over 150,000 people confirmed to have the virus in the UK of which over 28,000 deaths have been attributed alone or in part to COVID-19.^1^ There has been a significant reduction in ophthalmic outpatient attendance, diagnostic procedures and elective surgery as a direct effect from the lockdown and social distancing guidance from the government.^2^ This has caused a shift in focus to emergency presentations and reorganization of ophthalmic services.^3^ In its strategy to deal with the increasing influx of patients to acute medical care services, NHS England issued advice to doctors across all specialties on redeployment, specifically advising where and what roles they should undertake to help ease pressure from outstretched services during the pandemic.^4^

The Academy of Medical Royal Colleges further provided guidance in March 2020 outlining the importance of ensuring the training, competency and supervision of doctors before deployment outside their usual specialty or areas of expertise.^5^ It emphasized the advice that no doctor should undertake any duty that they do not feel comfortable to perform. For many ophthalmologists, redeployment was an unprecedented event. Therefore, we performed a survey open to all grades of ophthalmologists across the UK, irrespective of whether they were redeployed or not, to evaluate the processes, capture the experience and identify both the positive and negative aspects of redeployment. The survey had four main objectives:

1. To identify the proportion of each grade of ophthalmologists that were redeployed.
2. To recognize the medical and non-medical tasks performed by ophthalmologists during redeployment.
3. To demonstrate the levels of anxiety in doctors before and after redeployment and identify the factors influencing this.
4. To evaluate if the appropriate personal protective equipment (PPE) was provided for their respective redeployed place of work as recommended by Public Health England (PHE).

## Methods

An online prospective survey was developed by a group of ophthalmologists across the authors’ trusts to collect data on redeployment based on established literature on good practice.^6^ The study was conducted with non-random sampling distributed to ophthalmologists across the country of all grades using a multitude of methods including email of established networks, social media, and through the ophthalmic trainee group representatives at the Royal College of Ophthalmologists (RCO). The survey was open to all ophthalmologists irrespective of whether they were redeployed or not. The respondents who responded as deployed were taken to a set of questions regarding their redeployment while those that reported that they were not deployed answered a shorter survey regarding future redeployment. Not all responses were completed fully and although these were included in our study, only the answers they completed were considered in the analysis.

Responses were collected over a 2-week period between the 17th April 2020 and 1st May 2020 and the questionnaire was designed to be short and easy to complete. The survey was collected using an online provider who was able to prevent reduplication of the survey from the same device. Content was based around the NHS England guidance on “Redeployment of secondary care medical force safely.”^4^ The questions were designed to identify the impact of the current Coronavirus disease related redeployment on ophthalmologists to areas of need. Some of the questions assessed the PHE guidance issued on the 9th April 2020 on appropriate use of PPE for healthcare workers in different settings. We collected the responses according to their respective grades. In the UK, ophthalmology training is usually a 7-year process of specialty training (ST) progressing from year 1 (ST1) to year 7 (ST7). Responses from ophthalmology consultants, staff grades, associate specialists, specialty doctors and clinical fellows were also collected. Prior to survey distribution, we conducted a pilot questionnaire on a sample of ophthalmologists to ensure that it was quick and relevant for the purposes of our study. We also used some Likert-type questions to gain information from respondents about their anxieties before and during redeployment. This is a well-established method of collating information for surveys in healthcare and medical education.^7^

The data were assessed using the Shapiro–Wilk test, and found to be non-normally distributed. Hence, data are primarily reported as medians and interquartile ranges (IQRs) throughout. We used Mann–Whitney *U* tests for continuous and ordinal variables, and Fisher’s exact test and Chi-Squared for nominal variables. Pre- and post-deployment anxiety levels were paired for responses and compared using Wilcoxon’s signed rank test.

Analyses were performed using STATA® (StataCorp. 2015) and SPSS Statistics for Windows, Version 25.0 (IBM Corp, Armonk NY). Statistical significance was defined as *p* < 0.05.

This survey did not require ethics committee approval as it does not contain data from animal studies or human subjects.

## Results

In total, 145 surveys were returned and of these responses, 52% of respondents reported that they were redeployed. The respondents were categorized into four groups and the basic characteristics of each group are shown in Table 1. The trainee ophthalmologists were categorized to those with more recent experience of medical training, (ST1 & ST2 level) and those in ST3 and above. The group “Other” consisted of non-consultant career grade doctors such as staff grades, associate specialists, specialty doctors, and clinical fellows. The final group consisted of ophthalmology consultants irrespective of whether they were substantive or part time.

**Table 1.** Location of redeployment across all grades of ophthalmologists.

	All grades	Consultant	ST1-2 trainee	ST3+ trainee	Other (staff grade, fellow)
Total responses (%)	145 (100%)	24 (17%)	28 (19%)	73 (50%)	20 (14%)
**Deployed respondents**					
n (% of total deployed)	76 (100%)	7 (9%)	21 (28%)	39 (51%)	9 (12%)
Fully Completed Surveys n (%)	58 (76%)	7 (100%)	12 (57%)	31 (79%)	8 (89%)
**Non-deployed respondents**					
n (% of total non-deployed)	69 (100%)	17 (25%)	7 (10%)	34 (49%)	11 (16%)
Fully completed surveys n (%)	62 (90%)	16 (94%)	6 (86%)	31 (91%)	9 (81%)

While on redeployment, 74% of doctors reported that they did not perform any ophthalmic work.The remainder reported they participated in the ophthalmology on-calls (14%) or eye casualty shifts (10%). Half of the redeployed ophthalmologists (56.4%) performed 12-h shifts with or without night shifts or weekend shifts while the rest performed a resident 8-h shift during normal working hours.

## Location of redeployment

Table 2 outlines where each category of ophthalmologists was redeployed. Most doctors (84%) were redeployed to areas where patients with Coronavirus disease (COVID-19) were suspected or being treated. This included COVID-19 wards (50%), intensive care unit (ICU, 31%) and the emergency department (3%). No statistical significance was found between grade of doctor and location of redeployment (*p* = 0.198).

**Table 2.** Location of redeployment for all doctors who were redeployed.

	All grades	Consultant	ST1-2	ST3+	Other
	(*n* = 58)	(*n* = 7)	(*n* = 12)	(*n* = 31)	(*n* = 8)
COVID-19 medical/surgical ward	29 (50%)	5 (71%)	7 (58%)	13 (42%)	4 (50%)
NON COVID-19 medical surgical ward	8 (14%)	0 (0%)	3 (25%)	4 (13%)	1 (13%)
ITU/ICU	18 (31%)	1 (14%)	1 (8%)	13 (42%)	3 (38%)
Emergency department	2 (3%)	0 (0%)	1 (8%)	1 (3%)	0 (0%)
Other	1 (2%)	1 (14%)	0 (0%)	0 (0%)	0 (0%)

## Tasks performed during redeployment

In the survey, 60% of respondents felt they had adequate training regarding the tasks expected of them before starting their role in redeployment. Table 3 lists the tasks that were performed by ophthalmology doctors by their respective category. Most doctors reported carrying out multiple tasks. The ST1-2 group reported involvement in all aspects of care with exceptions in tasks relating to manual handling and general supportive care of patients (8%). In contrast senior trainees and consultants contributed more toward supportive care of patients. ST1-2 compared to ST3+ doctors performed significantly more clinical reviews of patients and of investigations (*p* = 0.019 and *p* = 0.04 respectively). ST1-2 group compared to consultants performed significantly more prescribing of medication (*p* = 0.036).

**Table 3.** Duties performed by ophthalmologist by grade.

	All grades	Consultant	ST1-2	ST3+	Other	*p*-value
	(*n* = 58)	(*n* = 7)	(*n* = 12)	(*n* = 31)	(*n* = 8)	
Clinical review of patients	43 (74%)	5 (71%)	12 (100%)	20 (65%)	6 (75%)	0.126
Review of investigations (blood tests, radiology imaging)	45 (78%)	6 (86%)	12 (100%)	21 (68%)	6 (75%)	0.139
Medication prescribing	45 (78%)	4 (57%)	12 (100%)	24 (77%)	5 (63%)	0.102
Supportive care (proning, washing, dressing)	19 (33%)	2 (29%)	1 (8%)	13 (42%)	3 (38%)	0.206
Other ward jobs	38 (66%)	2 (29%)	11 (92%)	20 (65%)	5 (63%)	0.048
Ward round participation	44 (76%)	5 (71%)	11 (92%)	23 (74%)	5 (63%)	0.468

## Anxiety before and after deployment

The anxiety scores before redeployment were scored on a scale of 1-5 for each survey participant and this was compared to their anxiety scores after redeployment (Appendix 2). Of those who were redeployed, there was a statistically significant improvement in anxiety after redeployment with a median pre-redeployment anxiety rating of 4 (moderately anxious) (IQR 3-5, mean = 3.6) and post-redeployment median of 3 (equivocal) (IQR 2-4, mean = 2.8). The difference in anxiety was found to be statistically significant (*p* < 0.001, Wilcoxon ranking).

**Figure 1.**
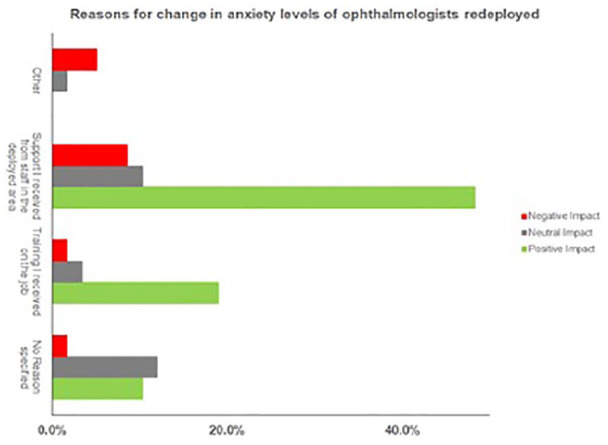
Graphical representation of the factors that impacted anxiety levels pre and post redeployment.

Fifty nine percent of people reported a reduction in anxiety with only 16% stating that their anxiety levels increased following redeployment. The main reasons for this are outlined in Figure 1 which illustrates that one of the main factors responsible for reducing anxiety was the support from the staff at the deployed area. Other reasons included concerns about the provision of adequate PPE, feeling out of their comfort zone and being in an environment with a high viral load.

For those who were not redeployed, 62 survey respondents reported a median of moderate anxiety at being redeployed. This was comparable to the anxiety level of the respondents who were redeployed with no statistically significant difference.

## Subgroup analysis of post deployment anxiety, confidence in tasks expected and clarity of responsibilities

We assessed the subjective questions of PPE availability and training adequacy on their respective post deployment anxiety scores, confidence in tasks expected and clarity of responsibilities. This is summarized in Table 4. Those that stated adequate provision of PPE were found to have a statistically significant reduced anxiety level and higher level of clarity of tasks expected from them following redeployment.

**Table 4.** Factors affecting anxiety, confidence in tasks and clarity on responsibilities during redeployment.

	Anxiety after deployment 1 (low) - 5 (high)		Confidence in tasks after deployment 1 (low) - 5 (high)		Clarity on responsibilities during redeployment 1 (low) - 5 (high)	
	Median (interquartile range)	*p*-value	Median (interquartile range)	*p*-value	Median (interquartile range)	*p*-value
**Those that responded PPE was adequate**						
No	4 (2-4)	**0.040**	1 (1-2)	0.059	2 (1-3)	**0.038**
Yes	3 (2-3)		2 (2-2)		3 (2-4)	
**Those that responded training was adequate (pre-deployment)**						
No	4 (2-4)	**0.006**	1 (1-2)	**<0.001**	2 (1-3)	**0.002**
Yes	3 (2-3)		2 (2-3)		3 (2-4)	
**Total**	**3 (2-4)**	**–**	**2 (1-2)**		**3 (2-3)**	**–**

Those that stated they had adequate training prior to redeployment were found to have a statistically significant reduced anxiety level, increased confidence in tasks during redeployment and higher level of clarity of tasks expected from them.

## PPE utilization during redeployment

As part of our survey, we evaluated the PPE provision of redeployed staff as advised by Public Health England (PHE) on hospital wards with or without confirmed or suspected Coronavirus disease, and for tasks that may be classed as an aerosol generating procedure. The guidance is shown in Appendix 1. We also specifically asked each survey respondent whether they felt that they had adequate PPE for the area they worked.

A total of 71% of survey respondents subjectively reported that they had adequate PPE for the area that they were assigned to. Table 5 illustrates the analysis of the PPE that was reported to be provided by each of the respondents. 71% of respondents were found to fulfill the specifications as published by Public Health England for the area they worked. The least adequate provision of PPE was for face/eye protection in wards with patients who had Coronovirus disease (59%).

**Table 5.** Reported PPE provision in different areas of redeployment.

	Eye/face protection	Respiratory protection	Hand protection	Body protection	Percentage fully met PPE criteria (PHE)
COVID Ward (*n* = 29) (%)	17 (59%)	27 (93%)	28 (97%)	27 (93%)	16 (55%)
ED (*n* = 2) (%)	2 (100%)	2 (100%)	2 (100%)	2 (100%)	2 (100%)
ITU/ICU (*n* = 18) (%)	16 (89%)	17 (94%)	18 (100%)	15 (89%)	15 (83%)
Non COVID (*n* = 9)	9 (100%)	9 (100%)	8 (89%)	9 (100%)	8 (89%)
ALL	44 (76%)	55 (95%)	56 (97%)	54 (93%)	41 (71%)

## Discussion

A novel coronavirus was identified by the Chinese Centre for Disease Control and Prevention following reports of pneumonia of unknown etiology in Wuhan, China in January 2020.^8^ It was termed COVID-19 and was declared a pandemic on 11 March by the WHO. The index case entered the UK on 23 January with subsequent rapid spread across the country bringing unprecedented pressure to the UK NHS.^9^ Significant reorganization and restructuring of hospitals and trusts became essential, with institutions focusing their efforts on life threatening presentations, in patients with Coronavirus disease. Our study has examined the extent and effect of redeployment on ophthalmology trainees to help with this crisis across the UK.

In the past, there have been cases where doctors were deployed to aid in a humanitarian cause such as the Ebola virus disease in West Africa or as military medics however there is nothing reported in the literature in the UK or other countries regarding a national scale redeployment across specialties. Currently there are a few emerging descriptive reports of changes in working pattern in COVID-19 where orthopedic surgeons were redeployed to the emergency room^10^ and the neurology department was re-structured to support the rest of the hospital to cope with COVID-19 related emergencies in the United States.^11^ In the UK, the NHS England has produced a guidance on “Redeploying your secondary care medical workforce safely” to support critical care services in pandemic, ophthalmology being one of the specialties to be redeployed.^4^ Ophthalmology represents a significant medical workforce in the UK. There are 135 NHS trusts which provide ophthalmic services with 1482 ophthalmic consultants, 662 staff grade, associate specialist and specialty grade doctors (SAS), and 706 ophthalmologists in training.^12^ Certainly in the authors’ trust, ophthalmology registrars and fellows are one of the few groups of doctors who have been redeployed to the ICU.

Our work is the first survey open to all ophthalmologists in the UK conducted to study redeployment to the unprecedented Coronavirus disease pandemic. From the survey respondents, 52% of ophthalmologists were redeployed across all grades, more than half of them being ST3+ ophthalmologists in training. The most common workplace was those areas with confirmed Coronavirus disease patients such as medical/surgical wards and ICU (81%). It was interesting to note that only two ophthalmologists (3%) were assigned to the ED. This is most likely to be due to the nature of ED working pattern where prompt management plan for new patients is required by all clinicians whereas ophthalmologists may be better utilized in a ward setting where they can support the medical team by performing other duties such as ordering investigations and prescribing. This is well shown in our survey that the main roles of ophthalmologists were review of investigations (78%), prescribing (78%) and clinical review of patients (74%). It is also worth noting that they were also able to perform clinical review of patients and this allowed the medical team to focus on tasks which require more complex medical decisions. Our results also demonstrated that senior ophthalmologists were more likely to be involved in supportive care than our junior colleagues (33% vs 8%) and junior colleagues were more involved in clinical care of patients (100% vs 74%). This is in accordance with the guidance published by the Academy of Medical Royal Colleges “Plans regarding trainee redeployment during the COVID-19 pandemic” which advocates skills-based redeployment of doctors.^5^ There was a statistically significant difference between the more junior ophthalmologists in training (ST 1-2) and their senior counterparts (ST3+) in the clinical review of patients and investigations. (*p* = 0.019 and *p* = 0.04 respectively). Naturally the younger ophthalmologists had more recent medical experience and were able to be more comfortable in these tasks compared to their senior counterparts.

Much anxiety was expressed among ophthalmologists and other doctors regarding redeployment.^13–15^ We have evaluated the anxiety level of ophthalmologists pre- and post-redeployment. A total of 59% of ophthalmologists reported improvement in anxiety level after the redeployment and this was statistically significant. A similar observation was made by other authors.^16^ This study identified that support received from the areas deployed and training had a significant effect on anxiety. It was also interesting to note that the anxiety levels were similar in both groups before redeployment (redeployment vs non-redeployment).

Our survey assessed the use of PPE in redeployed ophthalmologists. When we cross-checked the PPE requirement per location against the PHE guidance, we discovered that only 71% met the national criteria. This showed that the ophthalmologists were familiar enough with PHE guidance on appropriate PPE to identify shortfalls for the area they worked. The inadequate availability of PPE has been highlighted in the media to be a national problem initially in the UK.^17^ The study also showed that the largest proportion of PPE deficiencies were found in eye/face protection on wards with Coronavirus disease patients. This may be due to prioritization, frequent utilization and requirement with slow turnover of this kind of PPE. At the time of the survey, the requirement for eye protection was recently published and there may have been a delay in implementation & dissemination of PPE across the country. If we exclude eye/face protection, the compliance ranged from 83% to 100%. The British Medical Association (BMA) conducted a survey across specialties regarding PPE availability.^18^ Their results mirrored our findings among ophthalmologists, in fact some areas much worse. For example, in areas where aerosol-generating procedures were undertaken, 39.2% answered that they were provided with adequate eye/face protection in the BMA survey compared to our figure of 89% in ICU. In our study, the lowest provision was the eye/face protection in COVID ward (59%), compared to 35.05% in the BMA survey. A further study in the future assessing PPE provision would be useful to see if the initial shortfall of PPE compliance was addressed.

We recognize that there are some limitations to our study. Ophthalmologists who have been redeployed are more likely to respond to the survey compared to those who are not. Although our results showed that those who are not redeployed had a good survey completion rate, the questionnaire was shorter in this group. The response rate was low in the deployed ST1-2 group. This may be due to the fact that the majority of them were sent to wards (both COVID-19 and clean areas) where they were likely to have an antisocial shift pattern. There was a non-randomized distribution of the survey. This methodology was chosen to capture as many of those deployed across the UK targeting through trainee networks but in addition to allow snowballing of the survey from one respondent to another. This could have introduced a potential source of sampling bias. The survey allowed other reasons for anxiety to be identified such as the anxiety from litigation from working in an area outside of their expert area. We did note that none of the respondents mentioned this as a reason for their anxiety and this may be partly due to assurances from their trust of the provision of trust indemnity. Although the online survey provider only allowed a single response from a device, we do acknowledge that duplication of the survey could occur if an alternate device was used by the same individual. We have performed the survey at this point to capture the reflection in action, but we understand that it does not represent the overall view of the pandemic. Further studies assessing anxieties in other groups such as anesthetists or intensive care doctors would be useful to determine whether anxiety was created by being asked to work in unfamiliar physical environments with patients and diseases outside our usual practice or being in personal danger.

## Conclusions

We found that ophthalmologists of all grades contributed to support the frontline workforce in COVID-19 pandemic in the UK. There was much apprehension before redeployment, however we found that they were able to adapt to their new roles with appropriate support and training. PPE provision was mainly in keeping with guidance from Public Health England except for eye/face protection on wards with Coronavirus disease patients. This is the first time in our knowledge that ophthalmologists were redeployed to other specialties in a health pandemic. The results of this study should help reduce the anxiety of redeployed doctors in areas out of their normal expertise. The survey highlights and provides reassurance that at the time of need, stand-alone “specialist” services like ophthalmology can contribute to the work of the frontline staff in a supportive way outside their normal working practice.

## Supplemental Material

Appedix_1 – Supplemental material for Redeployment of ophthalmologists in the United Kingdom during the Coronavirus Disease Pandemic Click here for additional data file. Supplemental material, Appedix_1 for Redeployment of ophthalmologists in the United Kingdom during the Coronavirus Disease Pandemic by Christina Lim, Ian De Silva, George Moussa, Tahir Islam, Lina Osman, Huzaifa Malick, Sundeep Deol, Moheeb Youssef, Abdelsattar Farrag, Rehana Ashraf, Sreekala Burgula and Jonathan Thompson in European Journal of Ophthalmology

Appendix_2_1 – Supplemental material for Redeployment of ophthalmologists in the United Kingdom during the Coronavirus Disease Pandemic Click here for additional data file. Supplemental material, Appendix_2_1 for Redeployment of ophthalmologists in the United Kingdom during the Coronavirus Disease Pandemic by Christina Lim, Ian De Silva, George Moussa, Tahir Islam, Lina Osman, Huzaifa Malick, Sundeep Deol, Moheeb Youssef, Abdelsattar Farrag, Rehana Ashraf, Sreekala Burgula and Jonathan Thompson in European Journal of Ophthalmology
