# Supplementary figures and images for: Redeployment of ophthalmologists in the United Kingdom during the Coronavirus Disease Pandemic
Source: Eur J Ophthalmol. 2020 Aug 27;31(5):2268–74. doi: 10.1177/1120672120953339 (PMC7457008; doi:10.1177/1120672120953339)

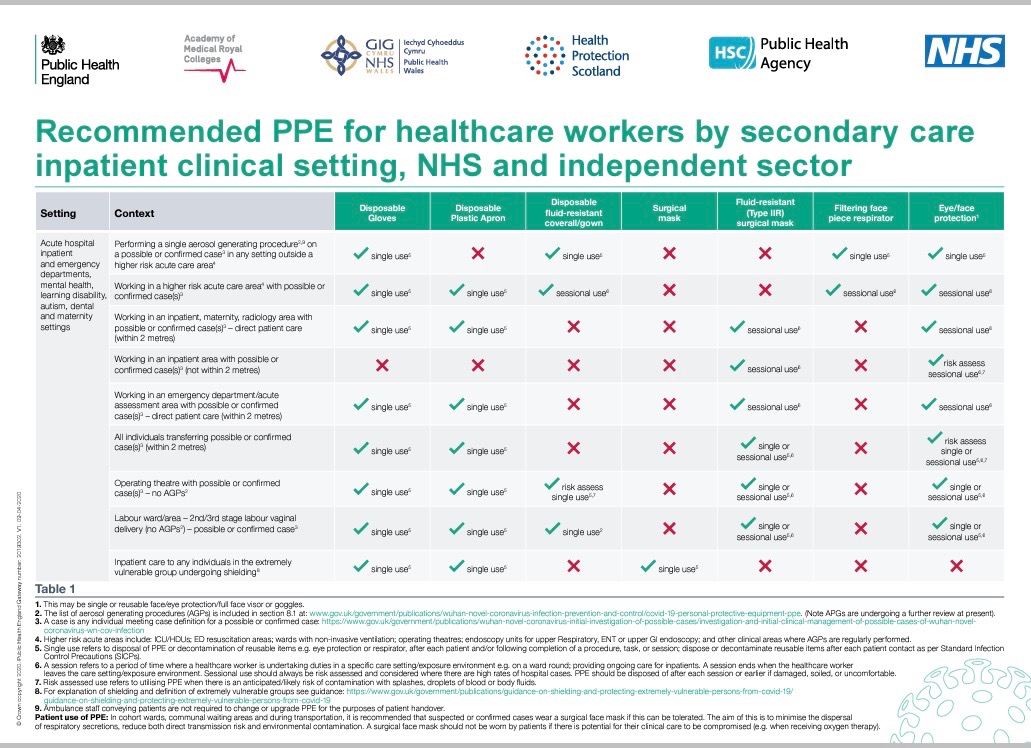

Supplement: Appedix_1 – Supplemental material for Redeployment of ophthalmologists in the United Kingdom during the Coronavirus Disease Pandemic [file Appedix_1.jpeg]
