# Supplementary material for: Redeployment of ophthalmologists in the United Kingdom during the Coronavirus Disease Pandemic
Source: Eur J Ophthalmol. 2020 Aug 27;31(5):2268–74. doi: 10.1177/1120672120953339 (PMC7457008; doi:10.1177/1120672120953339)
Supplement: Appendix_2_1 – Supplemental material for Redeployment of ophthalmologists in the United Kingdom during the Coronavirus Disease Pandemic [file Appendix_2_1.pdf]

Appendix 2

Nationwide Ophthalmology Redeployment Survey (NORS)

*** 1. What grade are you?**

ST 1 - ST 2

ST3 +

Post CCT Fellow

Consultant

Other (please specify)

*** 2. Have you been part or fully deployed to a service outside of ophthalmology during COVID 19?**

Yes	Go to Q 3
No	Go to Q 15

*** 3. Where were you redeployed to?**

ITU/ICU

COVID medical/surgical ward

NON-COVID medical/surgical ward

Emergency department

Other (please specify)

*** 4. During your time in redeployment, did you continue your duties in ophthalmology in any way? (please tick all that apply)**

No

Ophthalmology On-call

Ophthalmology eye casualty

Ophthalmology clinics/lasers/theatre

Ophthalmology admin

*** 5. In your time in redeployment, what shift pattern are you/were working? (please tick all that apply)**

Day (standard day shift)

Long days (12 hour shifts)

Nights

Ophthalmology Non resident on-call

On standby

*** 6. What PPE is provided in your redeployment area? (please tick all that apply)**

No Masks

Waterproof Surgical Mask

FFP2/ N95

FFP3

Full face respirator

No Gown

Plastic sleeveless aprons

Plastic full sleeved gowns

Surgical gowns

No Gloves

Single gloves

Double gloves

No eye protection

Additional eye protection (goggles, eye shields/spectacles)

*** 7. In your redeployment, do you feel that you had adequate training for your role (PPE, clinical tasks)?**

Yes

No

Comments

*** 8. Did you feel the PPE was adequate for your role in your redeployment area?**

Yes

No

9. What were/are your responsibilities during your redeployment? (please tick all that apply)

Clinical review of patients

Review of investigations

Medication prescribing

Supportive care (Proning, washing, dressing)

Ward jobs

Participate in ward round

Other (please specify)

***10. Whilst on redeployment, how clear are/ were you of your responsibilities? (5 – extremely clear, 1 – Not at all clear)**

Extremely clear

Fairly clear

Somewhat clear

Not so clear

Not at all clear

11. Generally, how confident did you feel in performing the tasks expected of you? (5 - a great deal confident, 1 - none at all)

A great deal

A Lot

A moderate amount

A Little

None at all

***12. Rate your anxiety level before and after redeployment**

	Not at all Anxious (1)	Little anxious (2)	Equivocal (3)	Moderately anxious (4)	Extremely anxious (5)
Before Deployment					
During/ After Deployment					

*** 13. What factors influenced the change in anxiety, (if applicable)? (Select more than one if applies)**

None

Training I received on the job

Support I received from staff in the deployed area

Other (please specify)

Survey END unless Q2 was answered No. If answer to Q2 was no then continue survey below.

*** 15. If not, are you aware that you will be deployed in the future?**

Yes

No

***16. How comfortable do you feel in terms of the tasks expected of you when deployed to an area outside of Ophthalmology?**

	Not at all Anxious (1)	Little anxious (2)	Equivocal (3)	Moderately anxious (4)	Extremely anxious (5)
Before Deployment					

***17. How comfortable do you feel about your own safety when you will be deployed to a service outside ophthalmology?**

	Not at all Anxious (1)	Little anxious (2)	Equivocal (3)	Moderately anxious (4)	Extremely anxious (5)
Before Deployment					

END OF SURVEY
